# Isolation and Phylogenetic Analysis of a Hunnivirus Strain in Water Buffaloes From China

**DOI:** 10.3389/fvets.2022.851743

**Published:** 2022-04-14

**Authors:** Haifeng Liu, Xinyue Zhu, Qinting Dong, Chengpeng Qiao, Yuhang Luo, Yi Liu, Yanlin Zou, Huanghao Liu, Cuilan Wu, Jieyu Su, Hao Peng, Kang Ouyang, Ying Chen, Jun Li, Zuzhang Wei, Weijian Huang

**Affiliations:** ^1^Laboratory of Animal Infectious Diseases and Molecular Immunology, College of Animal Science and Technology, Guangxi University, Nanning, China; ^2^Guangxi Key Laboratory of Veterinary Biotechnology, Guangxi Veterinary Research Institute, Nanning, China

**Keywords:** buffalo hunnivirus, MDBK cells, genome analysis, evolutionary analysis, isolation

## Abstract

In recent years, hunniviruses have been reported in a variety of animal species from many countries. Here, hunnivirus was detected in fecal samples from water buffaloes and named as BufHuV-GX-2106. The samples were inoculated into cultures of MDBK cells supplemented with TPCK trypsin and the BufHuV-GX-2106 strain was stably passaged and replicated. Electron microscopic analysis showed the BufHuV-GX-2106 virus particles were spherical and 20~30 nm in diameter. The complete genome of a plaque purified sample of BufHuV-GX-2106 was determined and analyzed. Genomic analysis revealed that the whole sequence of BufHuV-GX-2106 was ~7,601 nucleotides (nt) in length and consisted of a large open reading frame of 6,759nt, a 5′UTR, a 3'UTR and a poly(A) tail. The complete genome sequence of BufHuV-GX-2106 shares 68-85% nucleotide identities with other known hunnivirus strains, indicating high genetic heterogeneity among these viruses. Phylogenetic analysis showed that BufHuV-GX-2106 belonged to the *Hunnivirus A* species and was more closely related to ovine hunnivirus than other known viruses of this type. This study describes the first isolation and complete genome sequence of a hunnivirus strain from water buffaloes. In addition, this study will help to understand the mechanisms involved in the pathogenesis of *Hunnivirus A* among different animal species.

## Introduction

*Hunniviruses* are members of the *Hunnivirus* genus in the *Picornaviridae* family, in the order *Piccornavirales*. This genus contains a single species, *Hunnivirus A*. The genomic organization of hunnivirus is similar to that of other picornaviruses and has a linear genome ranging from 7.2 to 9.1 kb and contains a VPg, a 5′UTR, a large ORF encoding a single polyprotein, a 3′UTR and a poly(A) tail. The polyprotein encodes a leader protein (L) and functional regions P1, P2, and P3. The P1 region encodes the viral structural proteins (VP4-VP2-VP3-VP1), whereas the P2 region encodes the viral nonstructural proteins (2A^NPG↓*P*^-2B-2C) and the P3 region encodes nonstructural proteins (3A-3B^VPg^-3C^pro^-3D^pol^) (1).

Hunnivirus was first discovered from sheep cell cultures in Northern Ireland in 1965. In the last 20 years, hunniviruses have been reported in a variety of animal species from many countries, including fecal samples from healthy and diarrheic cattle and sheep in Hungary (2–4), rats in America (5), China (6, 7), Norway and Vietnam (8), as well as from diarrheic fecal samples from cats and pangolins in China (3). A high prevalence of rat hunnivirus (16%, 21/133) in Norwegian rats (*Rattus norvegicus*) in New York City, USA and bovine hunnivirus (15%, 4/26) in central Hungary was also reported. The prevalence of 17.8% of hunnivirus in *Rattus norvegicus* and 15.6% of hunnivirus in *Rattus tanezumi* were found in a total of 404 fecal samples collected from urban rats in Southern China, suggesting that hunniviruses are common in these urban animals. Nevertheless, information on the global distribution of hunniviruses in different animal species remains to be determined. Because there is a limitation of suitable cell culture systems and animal models for studying hunniviruses, the pathogenesis of these viruses is still unclear. In this study, we conducted a surveillance for buffalo hunnivirus in fecal samples obtained from two buffalo farms in Guangxi province, South China in 2021. We identified a novel hunnivirus in the diarrhea fecal samples of water buffaloes. This study describes the isolation and the genome characterization of this virus.

## Materials and Methods

### Sample Collection and Detection

A total of 198 fecal samples (38 diarrhea and 160 healthy fecal samples) from buffaloes were collected from two buffalo farms located in Nanning city, Guangxi Province, China in October 2020 to May 2021. The fecal samples were diluted with Dulbecco's Phosphate-Buffered Saline (DPBS) containing an antibiotic/antimycotic solution. The diluted samples were frozen and thawed out 3 times, followed by centrifugation at 12,000 rpm at 4°C for 10 min. 200 μl of the fecal supernatants was collected and stored at −40°C for RNA extraction and virus isolation. Viral nucleic acid was extracted by using an RNA extraction kit (AxyGen) according to the manufacturer's instructions. RT-PCR was then performed to detect hunniviruses using universal primers (UNIV-Kobu-F and UNIV-KobU-R) as described in a previous study (9). Thermal cycling conditions for each PCR fragment amplification were pre-denaturation at 98°C for 2 min, followed by 30 cycles of 95°C for 30 s, 58°C for 30 s, at 72°C for 45 s and a final elongation step at 72°C for 10 min. In the 198 fecal samples, other diarrhea related pathogens such as rotavirus, enterovirus, bovine virus diarrhea virus and bovine astroviruses were also investigated by using the methods as reported in previous studies (10–13).

### Cells and Antibody

MDBK, PK-15 and Vero cells were cultured in DMEM supplemented with 10% FBS. To generate the antibody against VP4 protein, the VP4 gene of BufHuV-GX-2106 was amplified by RT-PCR and cloned into pET-32a (+) expression vector (Novagen), resulting in a pET32a-VP4. The pET32a-VP4 was transformed into BL21(DE3) *Escherichia coli* cells. The cells were then induced by 0.1 mM IPTG for 4 h. The recombinant protein was purified using a HIS binding kit (Novagen). Polyclonal antibodies against BufHuv-VP4 protein were generated by injecting KunMing mice with the purified BufHuv-VP4 protein. This polyclonal antibody was purified by affinity chromatography with protein A.

### Virus Isolation

The fecal supernatants were filtered through 0.22 μm filters (Millipore, Billerica, MA, USA) and then stored at −80°C. MDBK, PK-15 and Vero cells were seeded in 12-well plate and these were inoculated with the filtered fecal supernatants. After 1 h of incubation at 37°C in an atmosphere of 5% CO_2_, the fecal supernatants were replaced with 2 ml DMEM containing 0.325 μg/ml TPCK treated trypsin (Sigma, St. Louis, MO, USA) and an antibiotic/antimycotic solution. The cells were maintained at 37°C in a 5% CO_2_ atmosphere and checked daily for the presence of cytopathic effects (CPE). After the appearance of CPE, the supernatants were harvested and then subsequently used for serial passages into MDBK cells.

### Complete Genome Determination

To determine the whole genome sequence of BufHuv-GX-2106, primers for PCR amplification were designed based on the genome sequence of OHuV-1 (Genbank accession no. HM153767). The primers used in this study are listed in Supplementary Table 1. Eight overlapping PCR fragments covering the whole genome were amplified by PCR with the specific primer sets. The PCR products were purified and cloned into pMD18-T vectors (TaKaRa, Dalian, China), followed by sequencing in both directions. Sequence assembly and analysis were performed by using DNAstar (Lansengene, Co) (14). The complete sequence obtained in this study was deposited to GenBank under accession no. OK642419.

### Plaque Assay and Plaques Purification

Viral plaque assays were performed using MDBK cells seeded in 6-well plates. MDBK cells were inoculated with 10-fold serially diluted viral samples and incubated at 37°C for 1 h. After washing with DPBS 3 times, the cells were overlaid with MEM containing 1% low melting agarose (Cambrex, Rockland, ME) incubated at 37°C for 48 h. After removal of the agarose, the cells were stained with 0.5% crystal violet and visible plaques were observed. In order to obtain a single viral clone, virus plaques purification were performed. Three viral plaques in the agarose were selected and then resuspended in DMEM by pipetting. After centrifugation, the supernatants were used to infect MDBK cells. The harvested viruses were serially passaged using MDBK cells.

### SDS-PAGE and Western Blot

The purified BufHuV-VP4 protein was transferred to polyvinylidene fluoride (PVDF) membrane by using 12% SDS-PAGE. PVDF membranes were blocked with 5% nonfat dry milk for 2 h at 37°C, and then incubated with monoclonal antibodies against the 6-His-tag overnight at 4°C. Subsequently, after washing five times with tris-buffered saline Tween-20 (TBST), the membranes were incubated with the secondary antibody HRP-goat anti-mouse IgG (H + L; 1:5,000) for 1 h at 37°C. After five washes with TBST, membrane-immobilized proteins were visualized using an enhanced chemiluminescence detection system.

### Indirect Immunofluorescence Assay (IFA)

MDBK cells were inoculated with BufHuV-GX-2106. At 48 hpi, the inoculum was discarded. The cell monolayers were triple washed with PBS and then fixed with cold acetone at −20°C for 30 min. After five washes with PBS, the cells were incubated at 37°C for 2 h a primary antibody, anti-BufHuV-VP4 PcAb (1:200). After five more washes with PBS, the cells were incubated with goat anti-mouse IgG H&L (Alexa Fluor^®^ 488, Proteintech), for 1 h at 37°C. Following five washes with PBS, the cell nuclei were visualized by using DAPI (blue) staining. Finally, images were captured under a fluorescence microscope whilst in PBS.

### Electron Microscope

Thirty ml of viral samples was centrifuged at 10,000 rpm at 4°C for 1 h. After centrifugation, the supernatants were filtered through 0.22 μm filters (Millipore, Billerica, MA, USA). Each filtered supernatant sample was harvested and mixed with 7.5 ml of 50% PEG-8000 reagent. After stirring at 4°C overnight, the mixture was centrifuged at 12,000 rpm at 4°C for 2 h. The supernatant was discarded after centrifugation and the precipitated viruses were resuspended in 1 ml of TBS. The virus particles were then negatively stained and was visualized by transmission electron microscopy.

### Virus Growth Curve

MDBK cells were inoculated with BufHuV-GX-2106 at a MOI of 0.1 and incubated at 37°C for 1 h. The MDBK cells were washed 3 times with DPBS, and cultured in 2 ml DMEM containing trypsin at a concentration of 0.325 μg/ml in a 5% CO_2_ incubator. Cell supernatants were collected at 6, 12, 24, 36, 48, 60, and 72 h, and stored at −80°C until used. The virus titers (TCID_50_) for each time point were assessed using MDBK cells and growth curves were calculated according to the Reed-Muench method.

### Sequence Alignments and Phylogenetic Analysis

The nucleotide sequences obtained in this study were aligned with published hunnivirus reference strains and representative picornaviruses by the ClustalW (1.6) method using DNAS Lasergene.v7 MegAlign and Editseq software. The information regarding representative picornaviruses is listed in Supplementary Table 2. Molecular evolutionary genetic analysis software (MEGA 5.0) was used to construct phylogenetic trees from the evolutionary distances obtained using the neighbor-joining (NJ) method. A GTR + G + I model was used to calculate genetic distances. P-distances for nucleotide sequences and the bootstrap test value were calculated by using 1,000 replicates, as described in previous studies (2, 15, 16).

## Results

### Isolation and Identification of a Buffalo Hunnivirus Strain

**Figure 1:**
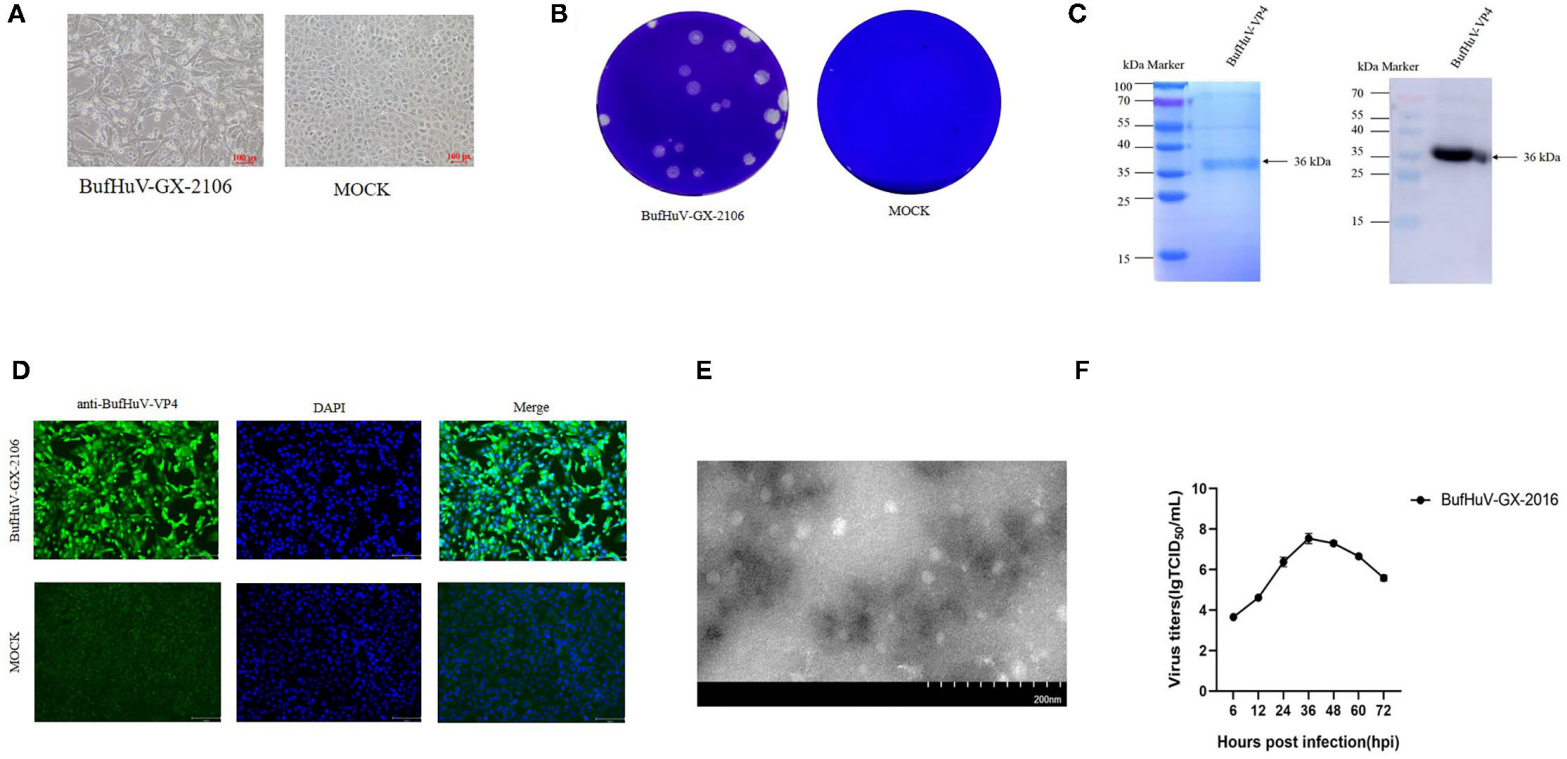
Isolation and identification of the BufHuV-GX-2106 strain in MDBK cells. **(A)** CPE in MDBK cells infected with the BufHuV-GX-2106 strain. Mock infected and virus infected MDBK cells were observed at 36 hpi. Photomicrographs were taken at a magnification of 20X. **(B)** Plaque morphology of the BufHuV-GX-2106 strain on MDBK cells. Monolayers of MDBK cells were inoculated with BufHuV-GX-2106 which were then overlaid with 1% agarose and stained with crystal violet at 48 hpi. **(C)** Analysis of expression of the purified BufHuV-VP4 by SDS-PAGE and western blot analysis of expressed recombinant BufHuV-VP4 showing reactivity with horseradish peroxidase-conjugated anti-His-tag monoclonal antibody. **(D)** IFA analysis of the VP4 protein expression was conducted in BHK-21 cells. The virus infected cells were fixed and stained using an anti-buffalo hunnivirus-VP4 PcAb and goat anti-rabbit IgG H&L (green). The cell nuclei were visualized by DAPI (blue) staining. **(E)** Electron microscopic examination of BufHuV-GX-2106 particles. MDBK cells were infected with BufHuV-GX-2106, and the precipitated viruses were processed for electron microscopy. **(F)** The growth of BufHuV-GX-2106 in cultured MDBK cells. At different times post infection, the cell supernatants were collected and the virus titers were determined as TCID_50_ values. The results represented are the means of three independent experiments.

A total of 198 fecal samples from two buffalo farms were collected and subjected to detection of hunniviruses. 1.5% (3/198) samples were positive for hunniviruses, as determined by RT-PCR (data not shown). The three positive samples belong to the same farm. In the 198 fecal samples, other diarrhea related pathogens such as rotavirus, enterovirus, bovine virus diarrhea virus and bovine astroviruses were also investigated. 4.5% (9/198) of fecal samples detected were positive for bovine astroviruses by RT-PCR (data not shown). The three fecal samples which were positive for hunnivirus were filtered and used for inoculation into MDBK cells, PK-15 cells and Vero cells. A typical CPE was only observed in MDBK cells inoculated with one of three samples after the 3rd passages (Figure 1A). The buffalo hunnivirus isolate, namely BufHuV-GX-2106, was obtained after a series plaque purification in MDBK cells. The plaques produced by the buffalo hunnivirus in MDBK cells were round in shape (Figure 1B). To generate the antibody against VP4 protein, the VP4 gene of BufHuV-GX-2106 was cloned into a pET-32a (+) expression vector and the recombinant clone was transformed into *E. coli* cells. The 36 kDa recombinant VP4 protein with a 6 his-tag was successfully expressed in *E. coli* strain, BL21(DE3). The VP4 protein was then purified using a HIS binding kit. The purified protein was confirmed by 12% SDS-PAGE and then analyzed by western blotting using monoclonal antibodies against the 6-His-tag, as shown in Figure 1C. Polyclonal antibodies against BufHuV-VP4 protein were generated by injecting KunMing mice with the purified BufHuV-VP4 protein._To confirm the isolation of hunnivirus, IFA analysis was conducted using a PcAb against BufHuV-VP4. As shown in (Figure 1D), MDBK cells infected with BufHuV-GX-2106 isolates reacted with specific polyclonal antibody raised against BufHuV VP4. The MDBK, PK-15 and Vero cell cultures without typical CPE were also analyzed by RT-PCR and IFA. Neither hunnivirus RNA nor proteins were detected in cell cultures (data no shown). Electron microscopic examination showed that BufHuV-GX-2106 strain particles were spherical and were 20~30 nm in diameter (Figure 1E). The multi-steps growth curves of the BufHuV-GX-2106 strain were assessed. Figure 1F shows the virus titers increased gradually from 6 hpi and reached the highest titers at 36 hpi and then dropped slowly after 48 hpi, suggesting that this virus grew well in cultured MDBK cells.

### Genomic Characteristics and Phylogenetic Analysis of the BufHuV-GX-2106 Strain

**Figure 2:**
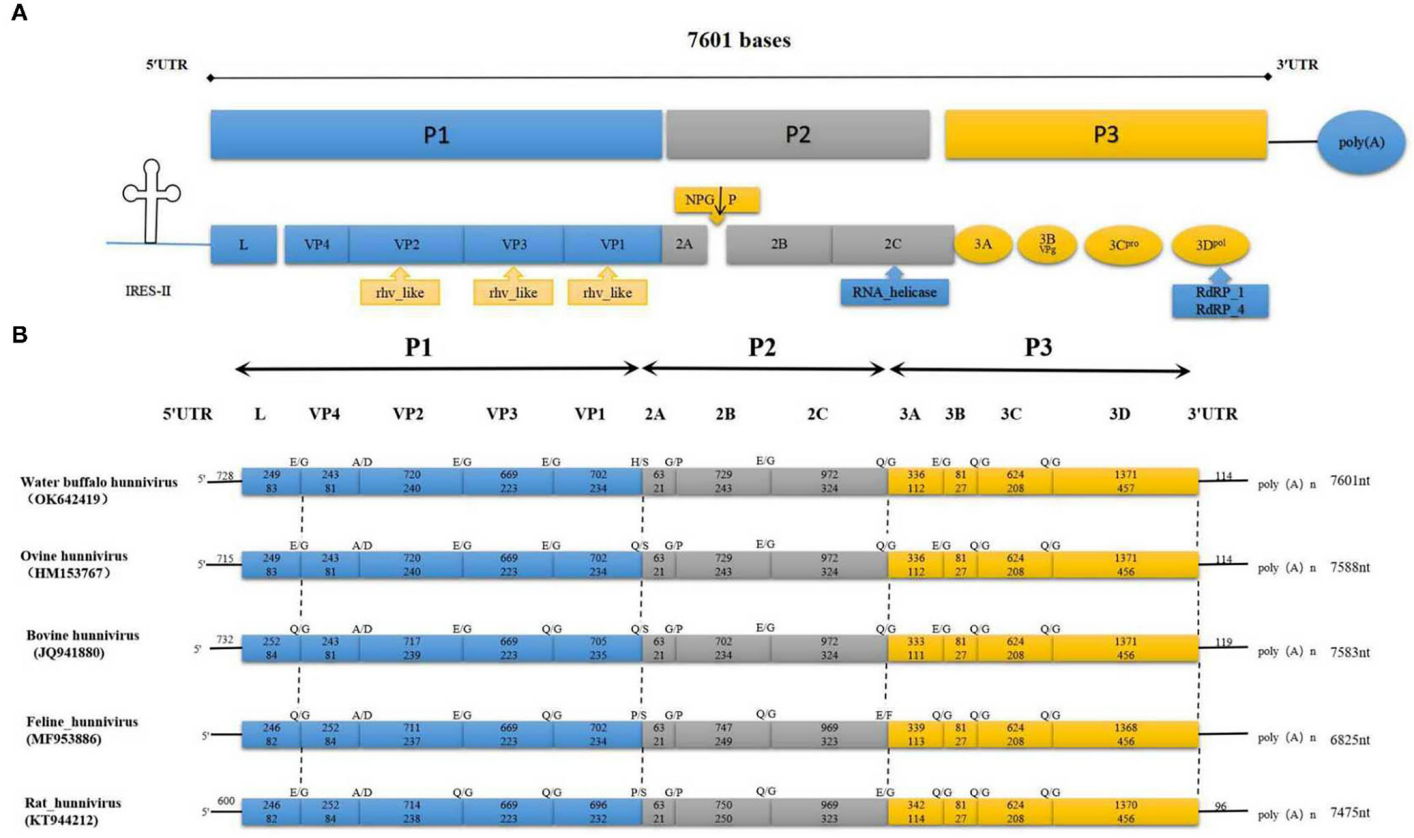
The complete genome and conserved motifs in the BufHuV-GX-2106 strain. **(A)** Diagrammatic representation of the complete genome consisting of a large ORF, 5′ UTR, 3′ UTR and poly A tail. P1 represents viral structural proteins and P2 and P3 represent non-structural proteins. The predicted conserved rhv-like domain, RNA helicase and RDRP in the large ORF coding region of BufHuV-GX-2106 are indicated adjacent to the arrows along the coding regions. **(B)** A comparison of the predicted cleavage sites among the referenced hunnivirus types. The predicted potential cleavage sites are, respectively, indicated above the arrows along the coding regions.

The entire sequence of BufHuV-GX-2106 was determined and analyzed. The complete genome sequence was submitted to GenBank and assigned an accession number, Ok642419. The entire genome of BufHuV-GX-2106 was found to be approximately 7,601 nt in length excluding the polyA tail. It possessed a typical picornavirus organization with a large open reading frame (ORF) of 6,759 nt, a 5′UTR of at the 5′-terminus, a 3′UTR and a poly(A) tail at the 3′-terminus, as shown in Figure 2A. The large ORF encodes a predicted polyprotein of 2,252 amino acids containing a structural protein, P1 (2,583 nt and 861 aa), non-structural proteins P2 (1,764 nt and 588 aa) and P3 (2,412 nt and 804 aa). The predicted genomic layout of BufHuV-GX-2106 was VPg+5′UTR-(L-VP4-VP2-VP3-VP1)-(2A^NPG↓*P*^-2B-2C)-(3A-3B^VPg^-3C^pro^-3D^pol^)-3′UTR-poly(A).

BufHuV-GX-2106 also has a putative rhv-like domain located at the VP1, VP2, and VP3 regions. The predicted RNA helicase and RDRP are located at 2C and 3D, respectively. Protease-cleavage sites within the polyprotein between the different types of hunniviruses were predicted by NetPicoRNA analysis and alignments with other known hunniviruses. As shown in Figure 2B, the predicted cleavage site for 2C/3A, 3B/3C and 3C/3D is Q/G. The predicted cleavage site for L/VP4, VP2/VP3, VP3/VP1, 2B/2C and 3A/3B is E/G. The predicted cleavage sites for VP4/VP2, VP1/2A and 2A/2B are A/D, H/S and G/P, respectively. All the predicted cleavage sites for each viral protein, except for VP1 and 2A of BufHuV-GX-2106 strain, are the same as those for ovine hunnivirus. In addition, the 5′UTR of BufHuV-GX-2106 has a putative type II IRES. As shown in Supplementary Figure 1, the conserved motifs I, J, K and L belonging to the type II IRES were located at positions nt 347–691 in 5′UTR. The predicted translation initiation site for the large ORF1 contains several optimal Kozak sequences. The polypyrimidine-rich region (UUUUCCUUUUU) is located 16 nucleotides upstream of the translation initiation site of ORF1. The Yn-Xm-AUG motif of BufHuV-GX-2106 was found to be Y11-X16-AUG.

The results of sequence comparison showed the complete genome of BufHuV-GX-2106 shares 68-85% identity with the referenced hunnivirus strains, and show the highest identity with ovine hunnivirus. The amino acid sequences of VP1, P1, P2, P3, 2C, and 3CD coded by BufHuV-GX-2106 ORF1 shares the highest 66, 69, 86, 92, 90, and 92% identities, respectively, with those of ovine hunnivirus. The details of the whole genome and the percentage of amino acid and sequence identities of the hunnivirus strains in this study are shown at Table 1.

**Table 1:** A comparative analysis of BufHuV-GX-2106 genome characteristics and other known hunnivirus genomes.

**Between species**	**Genomic characteristics**		**Amino acid identity (%)**						
			**BufHuV-GX-2106**						
	**Login ID**	**Length(n/t)**	**Complete genome**	**VP1**	**P1**	**P2**	**P3**	**2C**	**3CD**
Bovine_hunnivirus	NC_018668	7583	76	67	69	72	80	79	81
Ovine_hunnivirus	HM153767	7588	**85**	**66**	**68**	**86**	**92**	**90**	**92**
Feline_hunnivirus	MF953886	6825	69	62	59	63	75	71	76
Rat_hunnivirus	KT944212	7475	68	55	54	62	75	73	77

“*Homology comparison of amino acids based on the complete genome, VP1, P1, P2, P3, 2C, and 3CD. The highest identity (%) are marked in “bold.”*

**Figure 3:**
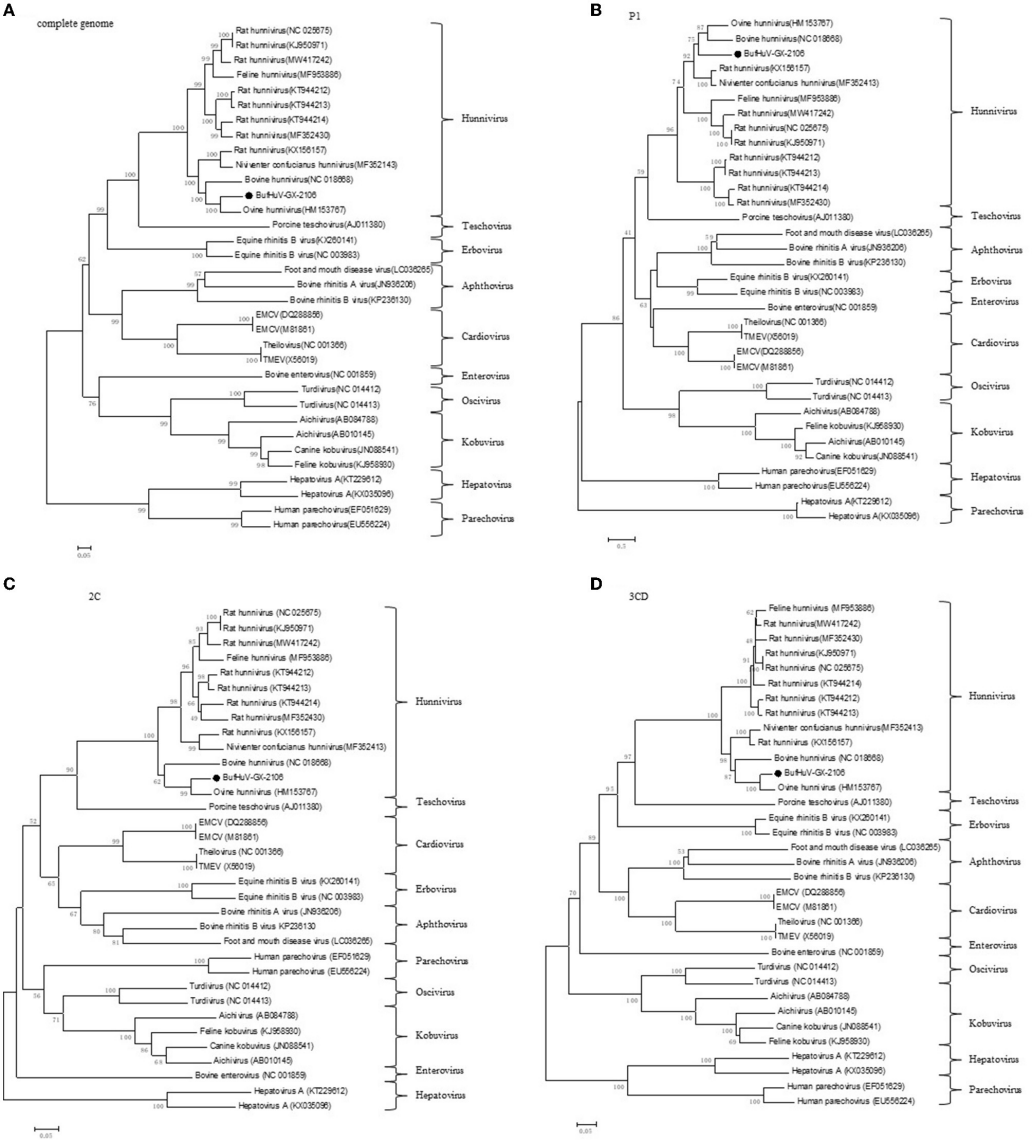
Phylogenetic analysis of BufHuV-GX-2106 with representative picornaviruses. Phylogenetic tree based on the complete genome sequence **(A)**, complete amino acid sequence of coding regions of P1 **(B)**, 2C **(C)** and 3CD **(D)** of BufHuV-GX-2106 and representative picornaviruses. The phylogenetic trees were generated using the neighbor-joining method implemented in the program MEGA5.0. Bootstrap values are expressed as a percentage based on 1,000 replications. The strain obtained in this study is indicated by the black dots.

Phylogenetic trees were constructed based on the complete genome and the complete amino acid coding sequences for P1, 2C, and 3CD of BufHuV-GX-2106 and those from representative picornaviruses. The results showed that BufHuV-GX-2106 was located in the same group as the members of the genus *Hunnivirus* in the *Picornaviridae* family in all four phylogenetic trees constructed (Figure 3). Consistent with the aa identities, BufHuV-GX-2106 was more closely related to OHuV-1 in the 2C, and 3CD proteins tree than other hunnivirus types. However, the P1 phylogenetic tree placed equidistantly BufHuV-GX-2106 closer to both the bovine hunnivirus (NC_018668) and ovine hunnivirus (HM153767) than to other hunniviruses.

## Discussion

Several hunnivirus strains were detected from a variety of animal species (cattle, sheep, rats, cats, pangolins) (2–8) from many countries. In the present study, we used the universal primers (UNIV-kobu-F and UNIV-kobu-R) to detect hunnivirus in the fecal samples of water buffaloes. Of the 198 fecal samples collected from two buffalo farms, 1.5% (3/198) were positive for hunnivirus, as determined by specific RT-PCR. Except 4.5% (9/198) positive samples for bovine astroviruses, the other diarrhea related pathogens such as rotavirus, enterovirus and bovine virus diarrhea virus were negatively determined by RT-PCR. The early hunnivirus strains were identified in healthy young domestic animals (2). A more recent study showed a hunnivirus strain could be detected from diarrheal stools of cattle and cats (3). The close relationship between hunnivirus infections and their clinical implications still remain uncertain. Further epidemiological, clinical and molecular studies are needed to investigate the geographic distribution, genetic diversity, host range and clinical importance of hunniviruses.

Like some enteric viruses, hunniviruses are difficult to propagate in conventional continuous cultured cell lines (2, 3). There are no reports on the successful isolation of hunnivirus strains. Trypsin appears to be an important co-factor needed for infectivity of these viruses in cell cultures. In the present study, we tried to grow the viruses in PK-15 and Vero cells, which have been previously used for virus isolation, in order to isolate hunniviruses. A typical CPE was only observed in MDBK cells inoculated with one of the three samples. After inoculating with the filtered fecal supernatants, MDBK cells were cultured with DMEM containing different concentrations of trypsin (0, 0.325, 0.6, 1, 2, 3, 5, and 6 μg/ml). No CPE was observed in MDBK cells when trypsin was absent in the culture medium. A stable cytopathic hunnivirus strain was isolated from cultured MDBK cells by adding a range of trypsin concentrations (0.325-6 μg/ml) of trypsin to maintain the culture medium. This provides a useful method for the isolation of hunniviruses. In addition, the successful isolation of this hunnivirus strain will help to understand the mechanisms involved in the pathogenesis of hunniviruses.

The whole genome sequence of the BufHuv-GX-2106 hunnivirus strain had a typical picornavirus organization with a large ORF, a 5′UTR at the 5′-terminus, a 3′UTR and poly(A) tail at the 3′-terminus. Because the 5′ and 3′-ends of the genome of BufHuv-GX-2106 hunnivirus strain were not determined by the RACE method, the exact sequences of both ends of the genome were unknown. Like the 5′ UTR of the other known hunniviruses, the 5′UTR of BufHuV-GX-2106 possesses a putative type II IRES bearing the conserved core-domain motifs I, J, K, and L. Similar to ovine and bovine hunniviruses (2, 17), the IRES of BufHuV-GX-2106 has a polypyrimidine-rich region (UUUUCCUUUUU) and this is located 16 nucleotides upstream of the AUG initiation site of ORF1. BufHuV-GX-2106 also has a putative rhv-like domain, RNA helicase and RDRP in the ORF1 coding region. BufHuV-GX-2106 also has predicated cleavage sites of the ORF1 coding protein for BufHuV-GX-2106 which are more similar to OhuV-1 than other hunnivirus types. A complete genome sequence comparison revealed that BufHuv-GX-2106 displayed the highest nucleotide identity (85%) to ovine hunnivirus and displayed rather low nucleotide identity (68-76%) to other known hunniviruses isolated from cattle, cats and rats. Consistent with the aa identities, phylogenetic tree analysis based on P1, 2C, 3CD and the complete genome indicated that BufHuV-GX-2106 was more closely related to OhuV-1 than any other hunnivirus types.

In summary, our study describes a reliable and optimized method for isolation of a novel hunnivirus strain from China. Complete sequencing of the hunnivirus, BufHuv-GX-2106, showed it to be the first strain isolated using established cell lines. In addition, BufHuv-GX-2106, is the first complete genome sequence of a cell-adapted hunnivirus strain known. These findings will be helpful for us to understand the genetic evolution of hunniviruses and provide the basis to further study the pathogenesis of these viruses.

## Data Availability Statement

The data that support the findings of this study are available from the corresponding author, ZW, zuzhangwei@gxu.edu.cn, upon reasonable request.

## Ethics Statement

The animal experiment protocols in this study were approved by the Ethics Committee of Guangxi University. Written informed consent was obtained from the owners for the participation of their animals in this study.

## Author Contributions

ZW: conceptualization. HaL, XZ, and QD: data curation. HaL, CQ, YuL, YiL, YZ, HuL, and CW: formal analysis. CW, JS, HP, KO, and YC: investigation. HaL and XZ: methodology. JL, ZW, and WH: project administration. ZW: writing—review and editing. All authors contributed to the article and approved the submitted version.

## Funding

This study was supported by the Integration and Demonstration of Technology for Improving the Quality and Efficiency of Dominant Characteristic Industries in Dashiishan District, Guangxi (Ministry of Science and Technology of the People's Republic of China grant no. 2021YFD1100100) and the Guangxi Key Laboratory of Veterinary Biotechnology Project (20-065-23-B-1).

## Conflict of Interest

The authors declare that the research was conducted in the absence of any commercial or financial relationships that could be construed as a potential conflict of interest.

## Publisher's Note

All claims expressed in this article are solely those of the authors and do not necessarily represent those of their affiliated organizations, or those of the publisher, the editors and the reviewers. Any product that may be evaluated in this article, or claim that may be made by its manufacturer, is not guaranteed or endorsed by the publisher.

## Supplementary Material

Supplementary Figure 1 The predicted RNA secondary structure of the IRES of BufHuV-GX-2106 strain. The 5′UTR of BufHuV-GX-2106 was predicted to possess a type II IRES. The conserved core-domain motifs I-J-K-L belonging to the type II IRES are indicated by the gray shaded boxes. The GRNA tetraloop motif, A bulge, polypyrimidine-rich region and predicted translation initiation site for the large ORF1 are indicated by the yellow shaded boxes.
