# Supplementary figures and images for: Isolation and Phylogenetic Analysis of a Hunnivirus Strain in Water Buffaloes From China
Source: Front Vet Sci. 2022 Apr 14;9:851743. doi: 10.3389/fvets.2022.851743 (PMC9047669; doi:10.3389/fvets.2022.851743)

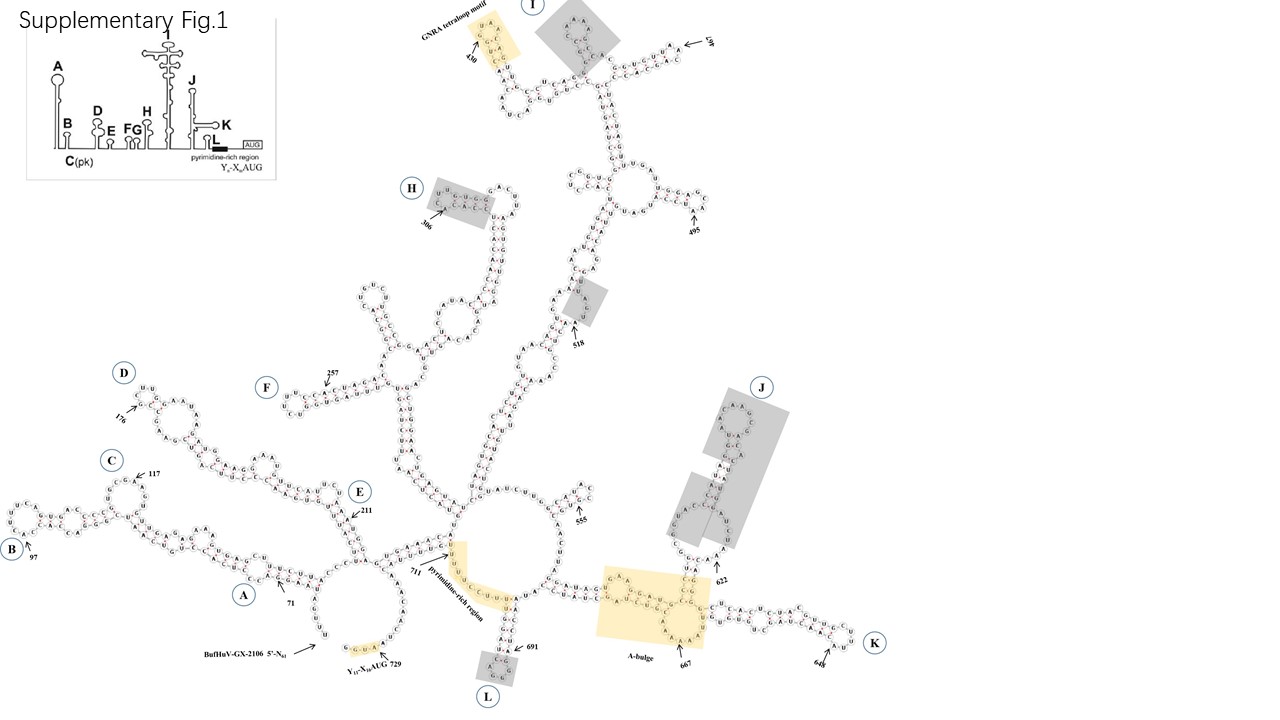

Supplement: Supplementary Figure 1 — The predicted RNA secondary structure of the IRES of BufHuV-GX-2106 strain. The 5′UTR of BufHuV-GX-2106 was predicted to possess a type II IRES. The conserved core-domain motifs I-J-K-L belonging to the type II IRES are indicated by the gray shaded boxes. The GRNA tetraloop motif, A bulge, polypyrimidine-rich region and predicted translation initiation site for the large ORF1 are indicated by the yellow shaded boxes. [file Image_1.JPEG]
