# Supplementary material for: Isolation and Phylogenetic Analysis of a Hunnivirus Strain in Water Buffaloes From China
Source: Front Vet Sci. 2022 Apr 14;9:851743. doi: 10.3389/fvets.2022.851743 (PMC9047669; doi:10.3389/fvets.2022.851743)
Supplement: Supplementary file 2 [file Table_1.docx]

Supplementary Table 1. The primer sequences used for BufHuV-GX-2106 in this study.

| Primer name | Sequence 5'-3' | Amplification length (bp) |
| --- | --- | --- |
| UNIV-kobu-R | ATGTTGTTRATGATGGTGTTGA | 216 |
| UNIV-kobu-F | TGGAYTACAAGTGTTTTGATGC |  |
| BufHuV-GX-2106-1-F | TATTTTCCCCTTTCCCT | 428 |
| BufHuV-GX-2106-429-R | CAACTGTTACCAGTTGT |  |
| BufHuV-GX-2106-80-F | CAATCGGGACCACCACTTTCAGTG | 1542 |
| BufHuV-GX-2106-1622-R | ATACACAAACTGGGGCACCA |  |
| BufHuV-GX-2106-1420-F | GTTCATCACACTGAAGATTGGTGA | 1090 |
| BufHuV-GX-2106-2510-R | CACTGCAAGTGTGGTCAATTGCCAT |  |
| BufHuV-GX-2106-2470-F | GAAGTTGGTAGTGGTGGGTATTTTAC | 1500 |
| BufHuV-GX-2106-3970-R | TCTTCTTGTGCCTGTTTGAT |  |
| BufHuV-GX-2106-3920-F | CCTAAGATTGACTACCCAGG | 1250 |
| BufHuV-GX-2106-5170-R | TGTGCGATTGCAAGGTCACA |  |
| BufHuV-GX-2106-4980-F | CGCCAGGTGTGAATGTAG | 1000 |
| BufHuV-GX-2106-5980-R | ACACTGCCACAAAACCCTGGTGCT |  |
| BufHuV-GX-2106-5934-F | ATGGCTAGCCGCAAACAGCA | 232 |
| BufHuV-GX-2106-6166-R | ATCCAACTGTACCGAGGGTTGT |  |
| BufHuV-GX-2106-6120-F | GCTGAATTCCAGGGGCGCAT | 960 |
| BufHuV-GX-2106-7080-R | TGAAACTCACCATCATACAC |  |
| BufHuV-GX-2106-6920-F | GCCTTGAAATATGTGGATTC | 700 |
| BufHuV-GX-2106-7620-R | GATTACTCTGGGGAAAATTA |  |
