# Supplementary material for: Isolation and Phylogenetic Analysis of a Hunnivirus Strain in Water Buffaloes From China
Source: Front Vet Sci. 2022 Apr 14;9:851743. doi: 10.3389/fvets.2022.851743 (PMC9047669; doi:10.3389/fvets.2022.851743)
Supplement: Supplementary file 3 [file Table_2.docx]

Supplementary Table 2. Information regarding the picornavirus strains which have been referenced in order to establish a genetic evolution analysis chart.

| Picornavirus genus | Virus (type) name | Genome features | | |
| --- | --- | --- | --- | --- |
|  |  | Genus | Accession no. | Size (nt) |
| *Kobuvirus* | Aichi virus | Human | AB010145 | 8251 |
|  | Aichi virus B | Bovine | AB084788 | 8374 |
|  | Canine kobuvirus | Canine | JN088541 | 8289 |
|  | Feline kobuvirus | Feline | KJ958930 | 8291 |
| *Aphthovirus* | Foot-and-mouth disease virus - type O | Bovine | LC036265 | 8216 |
|  | Bovine rhinitis B virus | Bovine | KP236130 | 7499 |
|  | Bovine rhinitis A virus | Bovine | JN936206 | 7250 |
| *Cardiovirus* | Encephalomyocarditis virus | Murine | DQ288856 | 7722 |
|  | Cardiovirus A1 | Human | M81861 | 7835 |
|  | Theilers murine Encephalomyelitis virus | Murine | X56019 | 8101 |
|  | Theilovirus | Murine | NC_001366 | 8101 |
| *Erbovirus* | Equine rhinitis B virus | Equine | KX260141 | 8823 |
|  |  | Equine | NC_003983 | 8828 |
| *Hepatovirus* | Hepatovirus A | Woodchuck | KT229612 | 7569 |
|  |  | Human | KX035096 | 7521 |
| *Parechovirus* | Human parechovirus 1 | Human | EF051629 | 7355 |
|  | Human parechovirus 7 | Human | EU556224 | 7146 |
| *Teschovirus* | Porcine enterovirus 1 | Porcine | AJ011380 | 7117 |
| *Oscivirus* | Turdivirus 2 | Oriental Magpie Robin | NC_014412 | 7641 |
|  | Turdivirus 3 | Grey-backed Thrush | NC_014413 | 7678 |
| *Enterovirus* | Bovine enterovirus | Bovine | NC_001859 | 7414 |
| *Hunnivirus* | Hunnivirus | Rattus | KT944213 | 7475 |
|  | Hunnivirus | Rattus | KT944212 | 7475 |
|  | Hunnivirus | Rattus | KT944214 | 7469 |
|  | Hunnivirus | Rattus | KX156157 | 7441 |
|  | Hunnivirus | Rattus | NC_025675 | 7496 |
|  | Hunnivirus | Rattus | MW417242 | 7282 |
|  | Hunnivirus | Rattus | MF352430 | 7626 |
|  | Hunnivirus | Rattus | KJ950971 | 7496 |
